# Prevalence and clonal lineages of biofilm-producing Staphylococcus aureus from clinical samples and healthcare workers at Ahmadu Bello University Teaching Hospital, Nigeria

**DOI:** 10.3205/dgkh000504

**Published:** 2024-10-23

**Authors:** Kabir Umar, Idris Nasir Abdullahi, Abdulkadir Magaji Magashi, Abdullahi Hassan Kawo, Yahaya Usman, Abdurrahaman El-fulaty Ahmad, Carmen Torres

**Affiliations:** 1 Department of Medical Laboratory Science, Faculty of Allied Health Sciences, Ahmadu Bello University, Zaria, Nigeria; 2 Area of Biochemistry and Molecular Biology, OneHealth-UR Research Group, University of La Rioja, Logroño, Spain; 3 Department of Microbiology, Faculty of Life Sciences, Bayero University, Kano, Nigeria

**Keywords:** biofilms, icaABCDR, panton-valentine leucocidin, healthcare-associated MRSA, toxic shock syndrome

## Abstract

This study determined the frequency and molecular features of *Staphylococcus aureus* from 206 burn and wound patients (BWPs) as well as 94 healthcare workers (HCWs) at the Ahmadu Bello University Teaching Hospital, Zaria, Northern Nigeria. Nine (4.4%) and five (5.3%) samples from BWPs and HCWs were identified as S*. aureus* positive, respectively. Seven (50%) were *mecA*-positive (associated with SCC*mec* types IVa and V), while 35.7% presented a multidrug resistance (MDR) phenotype. The *S. aureus* isolates belonged to 11 diverse *spa* types, including three new (t4539, t6043, t11694) and one singleton (t779), which were assigned to four clonal complexes. Two *tst* and three *luk-F/S-PV* carrying strains were identified. All the *S. aureus* isolates were moderate biofilm producers with diverse combinations of the *icaABCD* biofilm and *icaR* regulatory genes. The detection of genetically diverse *S. aureus* lineages and toxigenic strains highlights the need for improved surveillance of resistant and pathogenic strains in healthcare facilities.

## Abstract (de)

In der Studie wurden die Häufigkeit und die molekularen Merkmale von *Staphylococcus (S.) aureus* bei 206 Patienten mit Verbrennungen und Wunden sowie bei 94 Mitarbeitern des Ahmadu Bello University Teaching Hospital, Zaria, Nordnigeria, analysiert. In neun (4,4%) Proben von Verbrennungspatienten und fünf (5,3%) von medizinischem Personal wurde *S. aureus* nachgewiesen. Sieben (50%) waren *mecA*-positiv (assoziiert mit SCC*mec*-Typen IVa und V), 35,7% wiesen einen multiresistenten (MDR) Phänotyp auf. Die *S. aureus* Isolate gehörten zu 11 verschiedenen *Spa*-Typen, darunter drei neue (t4539, t6043, t11694) und ein Singleton (t779), die vier klonalen Komplexen zugeordnet wurden. Zwei *tst*- und drei *luk-F/S-PV*-tragende Stämme wurden identifiziert. Alle *S. aureus* Isolate waren mäßige Biofilmproduzenten mit verschiedenen Kombinationen der Biofilm- und *icaR*-Regulationsgene *icaABCD*. Der Nachweis genetisch unterschiedlicher *S. aureus*-Stämme und toxigener Stämme unterstreicht die Notwendigkeit einer verbesserten Überwachung resistenter und pathogener Stämme in Gesundheitseinrichtungen.

## Introduction

Burn and wound injuries are important causes of morbidity and death and are a common causes of hospitalization worldwide, necessitating both inpatient and outpatient care in many nations [1]. Bacterial infection accounts for significant morbidity and mortality in burn patients, and burn wound infection is the most common reason [2]. Burns and wounds provide a favorable site for bacterial multiplication and are more persistent, richer sources of infection than surgical wounds [3]. 

The bacteria that infect wounds and burn patients depend on multiple epidemiological factors. However, *Staphylococcus aureus*, a normal commensal in the nostrils and on skin, can be translocated to wounds and cause infectious processes [4]. Hence, *S. aureus* is one of the most frequently isolated pathogens in wounds and burns [5]. 

Healthcare-associated (HA)-MRSA is most frequently linked to a variety of infections in patients exposed to nosocomial settings. The emergence of community-associated (CA)-MRSA resulted in a significant shift in the epidemiology of MRSA isolates over the previous decades [4]. CA-MRSA can be distinguished by having distinct antimicrobial resistance patterns and molecular traits, although it is frequently identified by the lack of risk factors for HA-MRSA infections [6]. Generally, HA-MRSA typically harbours SCC*mec* I, II, and III, while CA-MRSA carries SCC*mec* IV or V [7]. Moreover, CA-MRSA isolates often carry the *lukSF-PV* genes that code for Panton-Valentine leukocidin (PVL), a cytolytic and toxic substance that has tropism for neutrophils [8]. 

The organism’s ability to spread rapidly through contact in the hospital ward poses a significant risk to managing burn and wound patients during hospital admission [9]. MRSA has been the center of concern due to its persistence and constant threat during the provision of healthcare services [10]. 

Moreover, healthcare workers (HCW) who frequently encounter sick individuals are at risk of contracting certain *S. aureus* strains from the patients and hospital environments. *S. aureus* is also often isolated from hospital curtains, surfaces, and equipment [11]. Hence, studying the nasal ecology of HCWs may provide greater insight into the potential transmission of pathogenic *S. aureus* strains in healthcare facilities. The treatment options for MRSA are becoming fewer by the day; this is associated with MRSA’s evolution into multi-drug resistance organisms, causing increased mortality around the globe [12]. Biofilm formation by MRSA worsens the situation by rendering it impenetrable, making the treatments more complex [13]. 

This study determined the frequency of *S. aureus* recovered from patients with burns and other wounds, as well as from healthcare workers at the Ahmadu Bello University Teaching Hospital, Zaria, Northern Nigeria. Moreover, the antimicrobial resistance (AMR) profile, biofilm formation capacity, biofilm and virulence genes, and lineages of the isolates were determined.

## Methods

### Study design and area

This cross-sectional study was conducted at Ahmadu Bello University Teaching Hospital Zaria, Kaduna State, on 206 burn- and wound patients (BWP) and 96 HCWs. The hospital is located in Shika, Zaria Local Government Area of Kaduna State, Nigeria. The 1000-bed capacity hospital serves as the main tertiary and reference hospital in the Northwest Geopolitical Zone. Approval (HREC number: ABUTHZ/HREC/W38/2020) was obtained from the Health Research Ethics Committee (HREC) of the Ahmadu Bello University Teaching Hospital, Zaria, before commencement of the study. All participants gave written informed consent before being recruited into this study.

### Sample collection 

The samples were collected aseptically. Swabs were taken from BWPs at sites with the highest deep-tissue exposure; the area was cleaned with sterile saline, after which the wound was swabbed. Moreover, a nasal swab was collected aseptically from HCWs (doctors, nurses, and hospital attendants) who were in contact with the BWPs. The samples were then transported to the Medical Microbiology Laboratory, ABUTH Zaria, for culture, bacterial isolation and identification. 

### Staphylococcus aureus identification 

Each sample collected was cultured on Mannitol Salt agar (MSA) and then incubated for 24 hours at 37°C. The isolates were identified using the following conventional biochemical tests: gram staining, growth patterns on MSA (yellow colonies), hemolysis on blood agar, catalase test, rabbit plasma coagulase test (slide test), and DNAse test. At the same time, resistance against cefoxitin (30 µg) was considered a positive test for MRSA by subjecting each organism to a sensitivity test using the Kirby-Bauer method. The Clinical and Laboratory Standards Institute (CLSI) guideline was used to determine resistance [14]. *S. aureus* identification was performed using mass spectrometry Matrix-Assisted Laser Desorption/Ionization Time-of-Flight (MALDI-TOF) using Biotyper software (Bruker) and the standard extraction protocol recommended by the manufacturer, as previously described [15]. Briefly, from a pure culture grown for 24 hours at 37°C in Brain heart infusion (BHI) agar medium, a small portion of the bacterial colony was transferred to the 96-well metal plate and left to dry at room temperature. Thereafter, the wells were covered with 1 µL of an alpha-cyano-4 hydroxycinnamic acid matrix (HCCA; Bruker). For the calibration of the spectrometer, the protein profile of the *E. coli* strain DH5 peptide was used. 

### Extraction of bacterial DNA 

For DNA extraction, the isolates were seeded on BHI agar and incubated for 24 h at 37°C. An isolated colony was suspended in 45 µL of sterile MiliQ water, then 5 µL of lysostaphin (1 mg/mL) (Sigma^®^) was added. The mixture was vortexed and incubated for 10 min at 37°C. Forty-five µL of sterile MiliQ water, 150 µL of Tris-HCl (0.1 M, pH 8) and 5 µL of proteinase K (2 mg/mL) (Sigma^®^) were added. This was vortexed and incubated for 10 min at 60°C. Finally, it was boiled for 5 min at 100°C and centrifuged at 12,000 rpm for 3 min. The DNA samples were stored at –20°C. The purity and concentration of the extracted DNA were measured using a NanoDrop UV-V is spectrophotometer (Thermo Fisher, USA).

### Antibiotic susceptibility testing 

Before the antibiotic susceptibility testing, the isolates were sub-cultured onto fresh nutrient agar slants incubated at 37°C for 24 hours. Suspensions were prepared from the sub-cultured isolates into clean, sterilized tubes using 0.5 McFarland’s standard. The isolates were then tested for their susceptibility to six antibiotics: penicillin G (10 IU), tetracycline (30 µg), erythromycin (15 µg), clindamycin (2 µg) doxycycline (30 µg), and levofloxacin (5 µg). The antibiotic discs (Oxoid, Thermo Fisher Scientific, Germany) were gently pressed to make sure they were in contact with the inoculated Mueller-Hinton agar surface, and the plates were incubated at 37°C for 24 hours. *S. aureus* ATCC 25923 was used as the control strain [16]. Zones of inhibition were measured to the nearest millimeter after incubation. The antibiotic breakpoints were determined using a chart adapted from the CLSI, 2022. Polymerase chain reaction (PCR) was conducted for the detection of the *mecA* gene from the *Staphylococcus aureus* isolates using the primers as presented in Table 1 (Tab. 1).

### Phenotypic detection of biofilm formation

A tissue culture plate (TCP) was carried out using the method described by Ansari et al. [13]. Ten milliliters of tryptic soy broth (TSB) with 1% glucose in test tubes were inoculated with a loop-full of test organisms from overnight culture on nutrient agar. The test tube was incubated at 37°C for 24 hours, and a dilution of 1:100 with fresh medium was made. After gentle mixing, the 96-well flat- bottom TCPs were filled with 0.2 mL of the diluted cultures. Sterile broth was used to serve as the blank. The culture plates were incubated at 37°C for 24 hours. After incubation, the microtiter plates were gently tapped. The wells were washed with 0.2 mL of phosphate buffer saline at pH 7.2 four times to remove free-floating bacteria. In contrast, the biofilm that remained adhered to the wells’ walls and bottoms was fixed with 2% sodium acetate and stained with 0.1% crystal violet. Excess stain was washed with deionized water, and plates were thoroughly dried. An optical density (OD) of stained adherent biofilm was obtained with a microtiter plate reader at a wavelength of 570 nm. The experiment was performed in 3x3. The OD cut-off (ODc) was calculated, which is the average OD values of the non-inoculated medium (ODN) plus 3x standard deviation of the non-inoculated medium (3xSD). The degree of biofilm formation was determined using the following Equations: 

**Equation 1:** OD<ODc: Non-biofilm producer.

**Equation 2: **OD≥ODc–OD<2xODc: Weak biofilm producer.

**Equation 3:** OD≥2xODc OD<4xODc: Moderate biofilm producer.

**Equation 4: **OD 4xODc: Strong biofilm producer.

### Detection of biofilm-associated and regulatory genes

The isolates were investigated for the presence of genes associated with biofilm formation, namely: *icaA*, *icaB,* *icaC,* *icaD,* and *icaR*. Using the method described by Mottola et al [17], the amplification reactions contain a mixture of 12.5 µl of Supreme NZYTaq 2x Green Master Mix (NZYTech, Portugal), 2 µl of each primer (forward and reverse) (STAB VIDA Lda, Portugal) and 6.5µl of sterile water (water for molecular biology, NZYTech, Portugal). Two µl of the previously extracted DNA was added to the mixture, resulting in a total reaction volume of 25 µl. PCR amplification was conducted in a thermal cycler (MyCycler Thermal Cycler, Bio-Rad, Portugal). 

### Molecular typing (staphylococcal protein A) 

All *S. aureus* strains were characterized by *spa* typing. After amplification of the hypervariable *spa* gene by PCR, Sanger sequencing of the amplicons was performed. For this, the variable fragment of the polymorphic region of the *spa* gene was amplified using forward and reverse primers (Table 1 (Tab. 1)) with the following cycling conditions: an initial denaturation at 95°C for 10 minutes followed by 35 cycles of denaturation at 95°C for 30 seconds, annealing at 60°C for 60 seconds, extension at 72°C for 45 seconds, and final extension at 72°C for 10 minutes [18]. After PCR, the amplicons were visualized on agarose gel via electrophoresis and viewed on a gel dock. The PCR products were then sequenced, and the sequence obtained was analyzed with the Ridom^®^ Staph-Type program (Ridom Gmbh; https://www.spaserver.ridom.de), which automatically assigns the *spa* type according to the repetitions detected. The clonal complex of the isolates was assigned according to the spa type, when possible.

### Detection of S. aureus toxigenic genes 

The *S. aureus* isolates were tested for the presence of *lukS-PV-lukF-PV* under the following cycling conditions: initial denaturation at 94°C for 5 minutes followed by 30 cycles of denaturation at 94°C for 30 seconds annealing at 55°C for 30 seconds, extension at 72°C for 1 minute and final extension at 72ºC for 10 minutes [19]. Moreover, all the strains were tested for the presence of the *tst* gene with a cycling condition of initial denaturation at 94°C for 5 minutes, followed by 30 cycles of denaturation at 94°C for 30 seconds, annealing at 55°C for 30 seconds, extension at 72ºC for 1 minute and final extension at 72°C for 10 minutes [20].

### Detection of staphylococcal complement inhibitor (scn) gene 

The MRSA and MSSA isolates were tested for the presence of the staphylococcal complement inhibitor *(scn)* gene with the following cycling conditions: initial denaturation at 95°C for 3 minutes, a series of denaturation at 94°C for 30 seconds, annealing at 53°C for 30 seconds for 30 cycles followed by extension at 72ºC for 2 minutes and final extension at 72ºC for 6 min.

## Results

### Demographic characteristics of the study participants

Of the three hundred participants, 206 (68.7%) were BWPs and 94 (31.3%) were HCWs. Males comprised 147 (49.0%) and females 153 (51.0%) of the study population. The median age of the study participants was 30.5 (IQR: 22–40) years. According to age categorization, the age group of 21–30 years was most highly represented with 90 (30.3%) participants, and the age group of 1–10 years was the least among the patients (4.7%). On the other hand, the study participants who were HCWs comprised 12 (4.0%) doctors, 52 (17.3%) nurses and 30 (10%) health assistants (Table 2 (Tab. 2)). 

### Prevalence of S. aureus among study participants 

**Figure 1:**
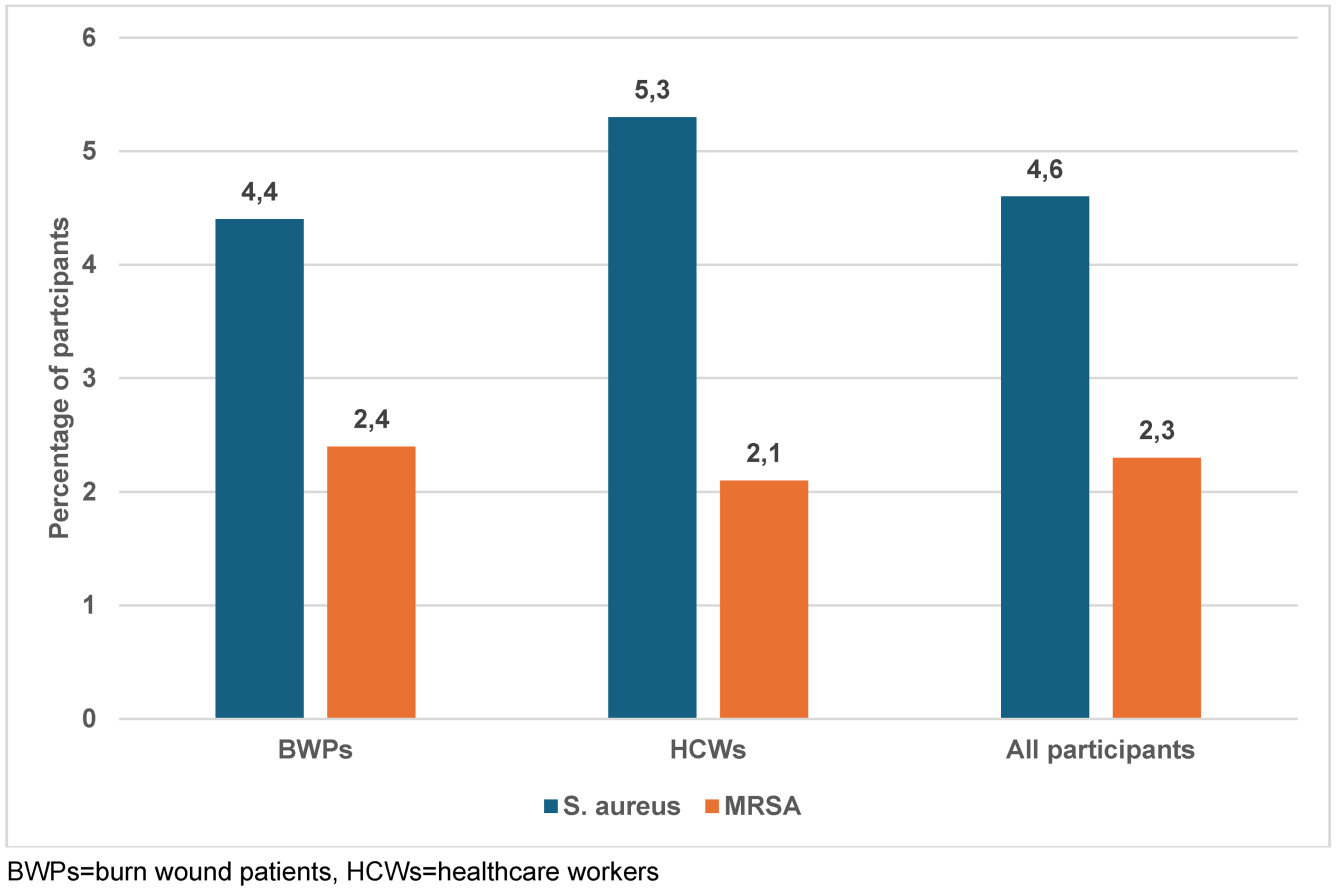
Frequency of *S. aureus* and MRSA carriage or infection among the study participants

A total of 68 (23%) samples were culture-positive on the Mannitol Salt Agar medium, and one isolate per sample was selected. After identification, only nine (4.4%) and five (5.3%) isolates from BWPs and HCWs consisted of *S. aureus*, respectively (Figure 1 (Fig. 1)). Eight (8) were *S. scuiri*, while *S waneri*, *S eqourum* and *S. heamolyticus* were found in one sample each. The *mecA* gene was detected in 7 (50%) (Table 3 (Tab. 3)). Of this, MRSA strains were detected in four BWPS (1.9%) and three HCWs (3.2%).

### Antimicrobial resistance and virulence profile of the S. aureus strains 

Seven *S. aureus* isolates (50%) were *mecA*-positive, two from BWPs and 5 from HCWs (associated with SCC*mec* types IVa and V), while 35.7% presented a multidrug resistance (MDR) phenotype. The following AMR phenotypes were obtained among *S. aureus* recovered from both HCWs and BWPs: penicillin (100%), levofloxacin (64.3%), doxycycline (50%), tetracycline (28.6%), erythromycin-clindamycin-constitutive (35.7%) and erythromycin (21.4%) (Table 3 (Tab. 3)).

The *S. aureus* isolates belonged to 11 different spa types, including three new (t4539, t6043 and t11694) and one singleton (t779), and were assigned to four clonal complexes (CC1, CC8, CC15 and CC88). Of these, CC88 (three MRSA and one MSSA) was the predominant complex (28.6%). The *S. aureus* lineages t064 and t127 came exclusively from HCWs, while t779 and t4539 were found in BWPs. Two *tst*-carrying strains (MRSA-t779 and MSSA-t779) and three *luk-F/S-PV* carrying strains (one MRSA-CC88-t786, MRSA-CC15-t084, MSSA-t4539) were identified. Some of the isolates (21.4%) were negative for the *scn* gene (human adaptation marker). All the *S. aureus* isolates were moderate biofilm producers with diverse combinations of the *icaABCD* biofilm and *icaR* regulatory genes (Table 4 (Tab. 4)). 

## Discussion

Methicillin-resistant *S. aureus* has been the center of concern due to its persistence and and constant threat during the provision of healthcare services [10]. Biofilm formation by MRSA worsens the situation by rendering it impenetrable, making treatment more complex [13]. Burn injuries are a significant health concern, particularly in resource-limited settings. Studying the characteristics and experiences of both patients and healthcare workers can contribute to better burn-care practices and interventions [21].

The overall prevalence of *S. aureus* was 4.6%, while that among HCWs and BWPs was 5.3% and 4.3% respectively, which is consistent with the finding of Gajdács et al. [22]. The lower sensitivity of the phenotypic method for identification of *S. aureus* could be due to the similarity of bacterial cultural features of *S. aureus* and other non-*aureu*s staphylococci, such as S*. haemolyticus* and *S. xylosus *[23]. Moreover, the agglutination method for the detection of coagulase enzyme could produce a false positive reaction [24], [25]. 

The overall prevalence of MRSA after *mecA* detection among recovered *S. aureus* was high (50%). This was lower than that obtained by Angel et al. [26] in Abuja Nigeria, who reported 93.8% *mecA*-positive isolate from patients, and higher than that obtained by Joshua et al. [27], who reported 15% in Zaria. Specifically, the low prevalence of *S. aureus* and MRSA identified from the BWPs in this study suggests that bacterial species other than *S. aureus* could have predominated in wound/burn infections [28]. In contrast, in the HCWs, the low frequencies of *S. aureus*/MRSA could be an indication of good infection control practices in the hospital. 

All MRSA isolates found in this study were phenotypically moderate biofilm producers, which was corroborated by genotypic detection of either of the biofilm-associated genes (ica) in all MRSA isolates. These findings are similar to the findings of Leshem et al. [29], who compared the ability to detect biofilm production of MSSA and MRSA by TCP and Congo Red methods. In addition, Silva et al. [30] reported agreement between biofilm detection through the TCP method and the detection of biofilm-associated genes. Similarly, Oche et al. [31] in Kano Nigeria reported a 94% detection rate of biofilm-producing *S. aureus* with a 100% biofilm-production rate of MRSA among orthopedic patients. The high rate of biofilm formation by MRSA could be linked to impaired wound healing due to the association of biofilm production and virulence of MRSA [32].

The tst gene that encodes toxic shock syndrome was detected in two isolates obtained from BWPs (one MRSA-t779 and one MSSA-t779). This is similar to finding of a study that reported the predominance of *tst*-producing *S. aureus-spa* type t779 lineage [33]. Contrary to our findings, Soltani et al. [34] reported that up to 18% of MRSA isolated from hospital settings carries the tst gene. However, *luk-F/S-PV*-carrying *S. aureus* strains were detected from both HCWs and BWPs. In this regard, the *luk-F/S-PV*-carrying MSSA strain was from only a BWP, while* luk-F/S-PV*-carrying MRSA strains were detected from a BWP and an HCW. These findings are similar to those previously reported by Joshua et al. [27] in Zaria and [35] in Abuja, in which they reported that about 10% of the *S. aureus* isolates carried the *luk-F/S-PV* gene. It is important to remark that all the participants who harbored the *tst* and *luk-F/S-PV* genes were BWPs. This could be because both virulence genes are predominantly associated with CA-MRSA, which suggests that patients might have contacted the organism even before admission [36]. All the toxigenic MRSA strains had SCC*mec* types (IVc and V), which denoted community acquisition.

Unlike tst and *luk-F/S-PV*, the *scn* gene (a host adaptation marker) was reported from both MRSA and MSSA. The presence of scn in most of the isolates suggests a potentially human origin [37]. The strains that were *scn*-negative could be due to loss of the sa3 prophage [38], which suggests animal origin and highlights potential zoonotic infection [39].

Diverse *S. aureus* lineages were identified, but the lineages CC15 and CC88 predominated in all participants. The MRSA-CC88 is termed the African Clone [40] and appears circulate widely in Nigeria [41], [42], [43]. It has been shown that the CC88 lineage is predominantly PVL-positive and spreads globally in hospital facilities [44], [45], [46], [47]. In this regard, the only MSSA-CC88 strain from the present study is *scn*-negative, which could support the previous findings. 

The lineage CC15 is ubiquitous and widely described in the literature, but these isolates are mostly MSSA and are often nasal colonizers [48]. Hence, the presence of this lineage in the BWPs indicates the translocation from the nose to the wound surfaces. 

Concerning the AMR profiles, relatively high resistance to fluoroquinolones, macrolide-lincosamide-streptogramins-B, and tetracycline were found. The macrolide and levofloxacin resistance was not surprising, as they among the top classes of antibiotics frequently prescribed in Nigeria [49]. However, tetracycline is a major antimicrobial agent of choice against *Staphylococcus* in both human and veterinary medicine [50]. Thus, resistance to these categories of antibiotics might be associated with high selective pressure due to their frequent use. 

### Limitation

Swabbing is not the gold standard for sampling from wounds; for instance, tissue biopsy or the Levine technique are more effective. Because of this, not all *S. aureus* could be recovered by swabbing. Other wound-associated bacterial pathogens could also be found in wound/burn patients. However, the focus of the present study was to investigate the potential contamination (nosocomial transmission) in either direction of wound patients or healthcare workers by the major nasal colonizer *S. aureus*. Thus, other bacteria were excluded. 

## Conclusions

A low frequency of *S. aureus* with less biofilm-producing ability was obtained. However, most of the isolates presented an MDR phenotype; a significant number of them were toxigenic. The results do not indicate a nosocomial event. However, the detection of diverse *S. aureus* lineages, resistance to first-line clinical antibiotics, and the ability of the virulent MRSA isolates highlight the need for improved surveillance of resistant and pathogenic strains in healthcare facilities. 

## Notes

### Competing interests

The authors declare that they have no competing interests.

### Data Availability Statement 

All the data derived from this study have been presented in this article. However, additional information may be requested from the corresponding author (Dr. Idris Nasir Abdullahi).

### Authors’ ORCID

- Umar K: 0000-0003-2500-2822
- Abdullahi IN: 0000-0002-5511-1272
- Usman Y: 0000-0003-3972-5351
- El-fulaty Ahmad A: 0000-0003-1941-8346
- Torres C: 0000-0003-3709-1690

### Funding

This study was funded by TetFund Nigeria through an institutional-based research grant (grant number: TETF/DR&D/UNI/ZARIA/IBR/2020/VOL.1/4).

## Figures and Tables

**Table 1:**
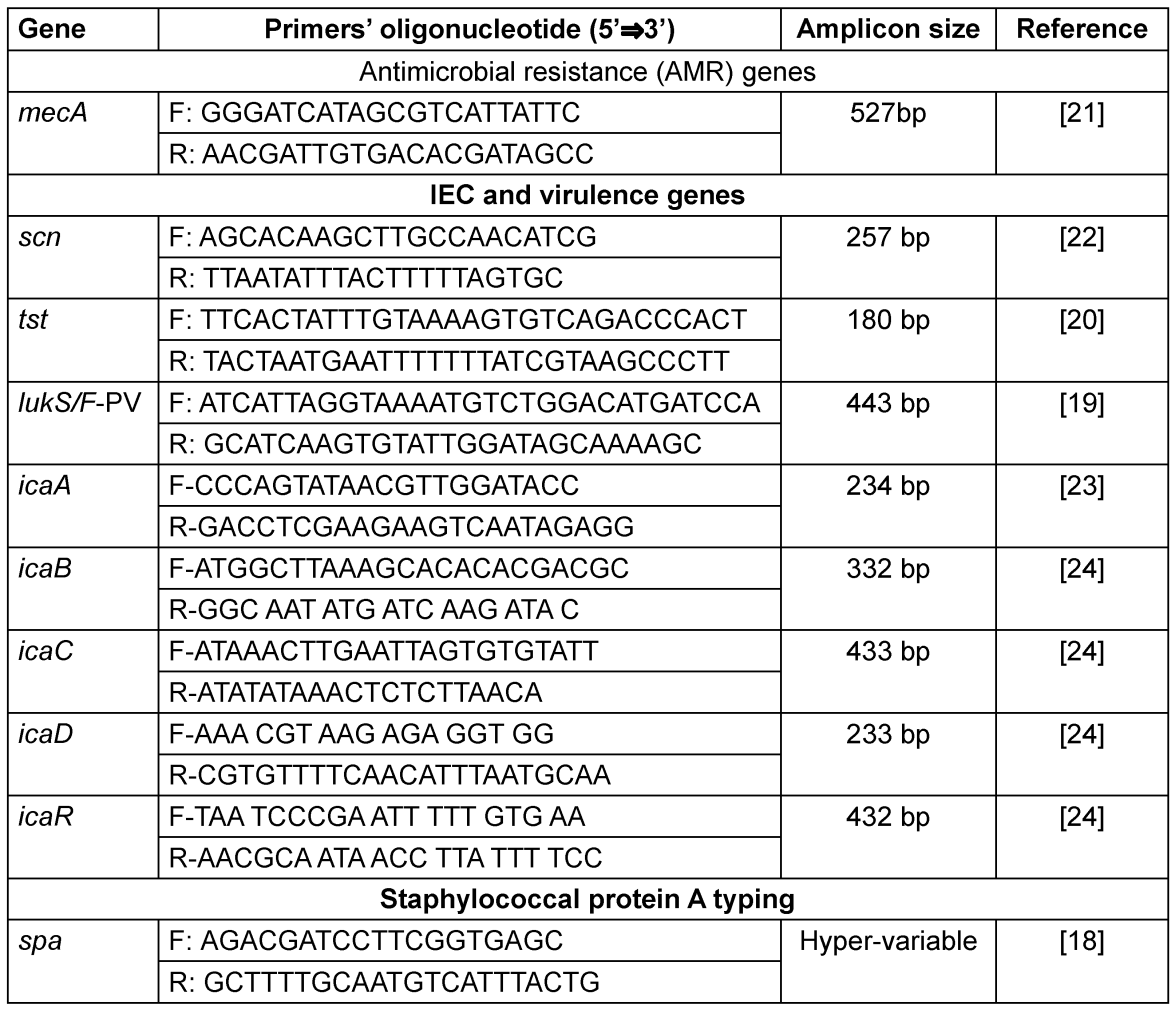
Genes and primer sequences utilized in all PCRs

**Table 2:**
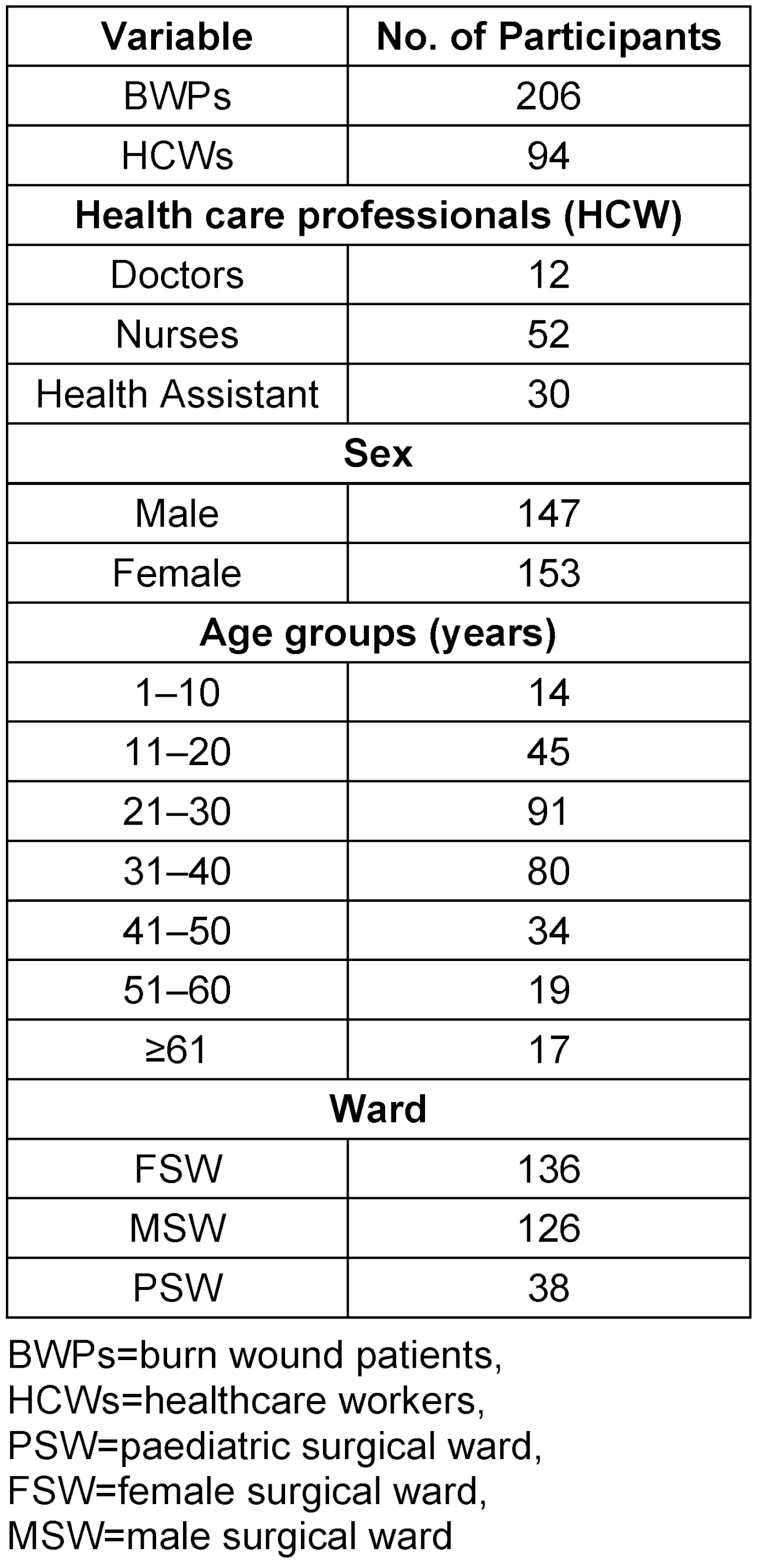
Socio-demographic characteristics of the participants

**Table 3:**
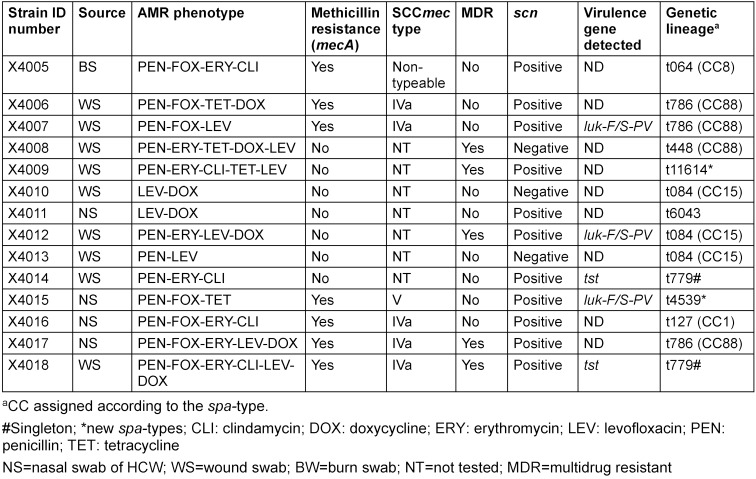
Molecular typing and antimicrobial resistance profile of the 14 *S. aureus* isolates from the study participants

**Table 4:**
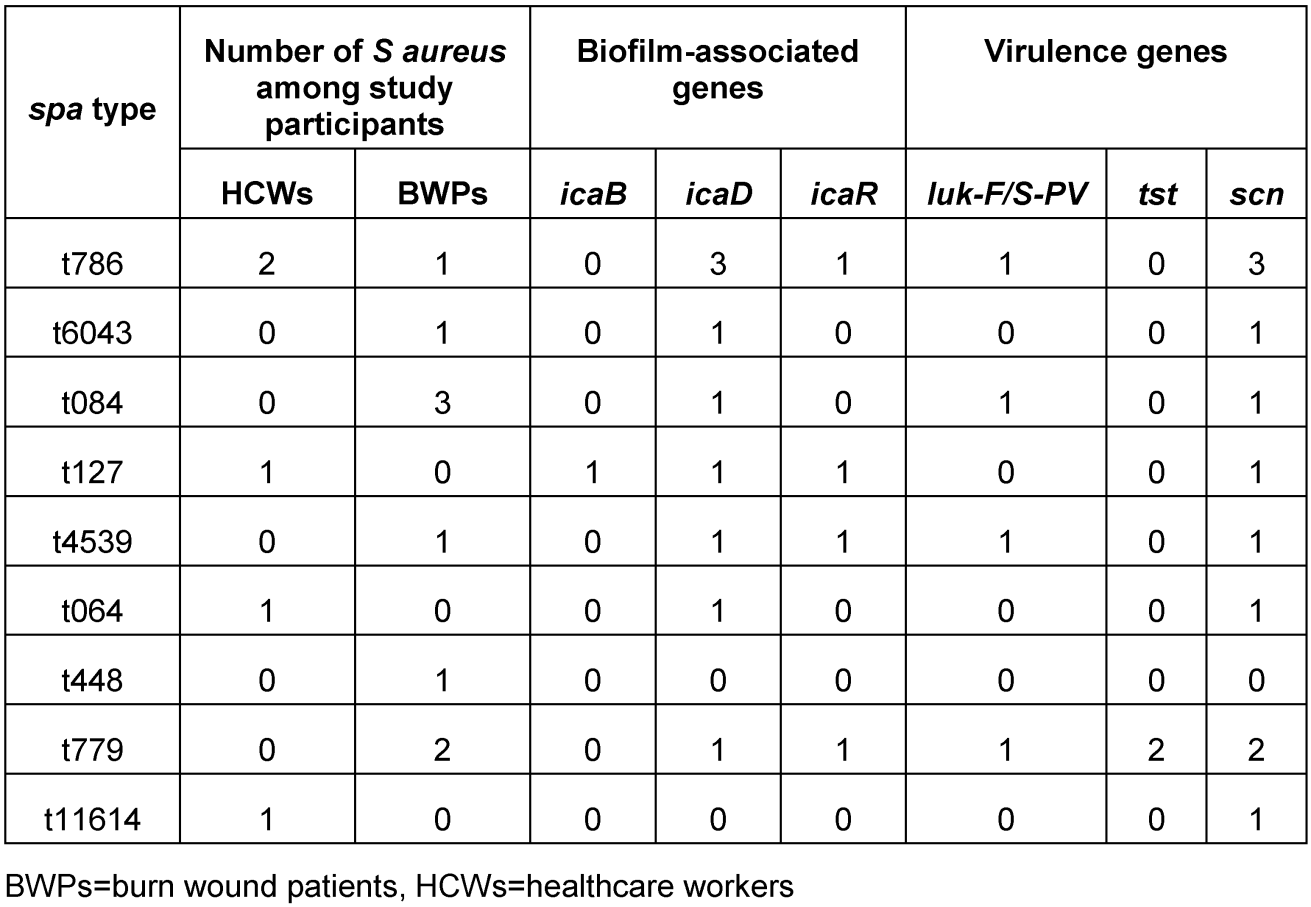
Molecular characteristics and virulence genes of the 14 *S. aureus* strains
